# 

**Published:** 1851-03

**Authors:** 


### NOTICE TO CORRESPONDENTS.

Communications and Books for notice should be addressed to the Editors, care of Messrs. Lindsay & Blakiston.

Letters, &c., connected with the *business affairs* of the Journal should be addressed to the Publishers.

Papers for publication must be received *before* the 20th of the month, or they cannot appear in the forthcoming number.

The following Journals have been received in exchange:

Medical News. Philadelphia.
Philadelphia Lancet.
New Hampshire Journal of Medicine.
New York Register of Medicine and Pharmacy.
New York Medical Gazette.
The Boston Medical and Surgical Journal. (Weekly.)
Buffalo Journal.
The London Lancet. (Weekly, London.)
The Medical Times. (Weekly, London.)
Dublin Medical Press.

---

The following works have also been received for notice:

Ricord's Illustrations of Venereal. From Mr. A. Hart.
Billings' Principles of Medicine. From Lea & Blanchard.
Murphy's Students' Review of Chemistry. From Lindsay & Blakiston.
Flagg on Ether and Chloroform. From the same.

---

Communications have been received from:—

G. W. Brown, M. D., Port Carbon, Va.
R. H. Smith, M. D., Dilworthtown, Pa.
W. Jewell, M. D., Philadelphia.
T. J. Gallaher, M. D., Pittsburgh, Pa.

---

The foreign correspondents of the Examiner will please direct their Exchanges and other communications to the care of Mr. Thos. Delf, 49 Bow Lane, London, or the Aldine Chambers, No. 13 Paternoster Row, or Mr. H. Bossange, 21 Bis, Quai Voltaire, Paris.

